# Biomimetic Wings for Micro Air Vehicles

**DOI:** 10.3390/biomimetics9090553

**Published:** 2024-09-14

**Authors:** Giorgio Moscato, Giovanni P. Romano

**Affiliations:** Department Mechanical & Aerospace Engineering, University La Sapienza, 00184 Roma, Italy; giorgio.moscato@uniroma1.it

**Keywords:** aerodynamics, micro air vehicles (MAVs), insects, wing corrugations, particle image velocimetry (PIV)

## Abstract

In this work, micro air vehicles (MAVs) equipped with bio-inspired wings are investigated experimentally in wind tunnel. The starting point is that insects such as dragonflies, butterflies and locusts have wings with rigid tubular elements (corrugation) connected by flexible parts (profiling). So far, it is important to understand the specific aerodynamic effects of corrugation and profiling as applied to conventional wings for the optimization of low-Reynolds-number aerodynamics. The present study, in comparison to previous investigations on the topic, considers whole MAVs rather than isolated wings. A planform with a low aperture-to-chord ratio is employed in order to investigate the interaction between large tip vortices and the flow over the wing surface at large angles of incidence. Comparisons are made by measuring global aerodynamic loads using force balance, specifically drag and lift, and detailed local velocity fields over wing surfaces, by means of particle image velocimetry (PIV). This type of combined global–local investigation allows describing and relating overall MAV performance to detailed high-resolution flow fields. The results indicate that the combination of wing corrugation and profiling gives effective enhancements in performance, around 50%, in comparison to the classical flat-plate configuration. These results are particularly relevant in the framework of low-aspect-ratio MAVs, undergoing beneficial interactions between tip vortices and large-scale separation.

## 1. Introduction

This work deals with aerodynamic effects due to bio-inspired corrugations on the wing surface of a micro air vehicle (MAV). These corrugations are inspired by those present on wings of several insects, as for example dragonfly or butterflies. The relevance of the present investigation lies in specific applications of bio-inspired insect wings to unmanned air vehicles (UAVs) and MAVs, considering that they can be also employed in small wind turbines, working at moderate Reynolds number intervals.

Among several possible flow control devices, the attention is here focused onto passive ones due to their simple manufacturing, continuous working operation and more precise computation of possible improvements in performance. Low-Reynolds-number passive control devices have received increased attention in the last twenty years, due to the sharp reduction in the aerodynamic performance of conventional wings for Reynolds numbers lower than 10^5^ [1]. The main source of performance degradation at low Reynolds numbers is the separation bubble on the wing upper surface, which increases drag and reduces lift [2]. So far, those airfoils, which have high performances at large Reynolds numbers, usually strongly degrade when used on small-scale MAVs [3]. On the other hand, in this flow regime, the use of passive rough elements placed at specific positions on the wing surface allows improving the wing efficiency by one order of magnitude [4].

One of the most recent advances in low-Reynolds-number aerodynamics is the use of devices recalling similarities with nature (bio-mimetics). Specific observations have been made regarding the wings of dragonflies, butterflies and locusts, by focusing on the fact that theirs wings are not smooth but rather show venations both longitudinally and transversally [5,6]. Moreover, their wings cannot be significantly deformed during flight by the combined action of muscles, as in flying mammals or in birds, and they do not have feathers. So far, mechanical structure investigations of these wings started from studies made by biologists [6], fascinated by such ultra-light wings [5]. These investigations were focused on improved structural mechanics analysis, stability and robustness [7,8,9], increasing load capacity and flying maneuverability [10]. In parallel, numerical and experimental investigations were carried out for testing the aerodynamic performance of wing configurations derived from this concept [11,12]. Among the different results, depending on the specific geometry employed and on the Reynolds number, there is general agreement that corrugated airfoils or wings could have similar or even better performance in comparison to conventional smooth-surfaced airfoils for Reynolds numbers around 10^5^ [10,11,12,13,14,15]. Attention has also been focused on wing configurations in tandem [13], where lift and drag were observed to be marginally affected by corrugation, and on flapping wings in unsteady conditions [12,14,16]. However, there are conditions and specific Reynolds number regimes in which the positive effects of corrugation on performance have been questioned [17], so that further investigations are surely required.

Previous investigations mostly focused on the effect of corrugations in airfoils or slender wing performances, as in real dragonflies [18,19,20]. However, for practical applications, testing on full MAVs equipped with corrugated wings would be desirable. This allows testing compact configurations, retaining the complexity of a full vehicle, rather than a single airfoil or wing. To this end, the analysis of corrugated wing performance should be extended to wings with a small aperture-to-chord ratio (aspect ratio, AR~1 or less), where tip vortices extended over the whole wing size [21,22].

Indeed, in low-aspect-ratio wings, lift is determined as the combination of the linear contribution due to circulation, as in airfoils, and the non-linear contribution due to the effect of tip vortices [21]. This second contribution is inversely dependent on wing aspect ratio, so it is particularly relevant in compact wing configurations, allowing them to generate lift at high angles of attack. It is expected that this would be highly beneficial for MAVs. The counterpart for lift enhancement is the parallel increase in drag due to the induced contribution, again related to tip vortex strength. This contribution is also dependent on the inverse of the aspect ratio, so it decreases with wing aperture. However, it is important to consider that the other additional drag term, parasitic drag, has a direct dependence on the aspect ratio, so that the overall drag contribution is both aspect ratio- and Reynolds number-dependent [22]. Surely, this is also of interest in evaluating corrugated wing-based MAV performance.

So far, it is of high practical interest to quantify corrugated wing improvements by applying possible solutions to real MAVs and to understand details of the flow field that are dependent on the specific geometry of corrugation. Therefore, starting from a previous investigation on bio-inspired corrugation, this paper aims to investigate a full MAV with a small aperture-to-chord ratio (aspect ratio, AR = 1) and a square planform at Reynolds numbers between 8 × 10^4^ and 1.3 × 10^5^ (based on the wing chord), which are typical of possible applications. It is important to specify that the corrugations investigated in the present study are orthogonal to the mean flow. Although, as stated previously, corrugations and venations in insects are present in all directions, most previous studies have considered corrugations aligned to the wind. The rationale to focus on transverse corrugations here is that this configuration will mostly affect large-scale separation, so it is surely more suitable to the objective of the present investigation regarding the interaction with large scale vortices generated in small-aspect-ratio MAVs.

## 2. Materials and Methods

**Figure 1:**
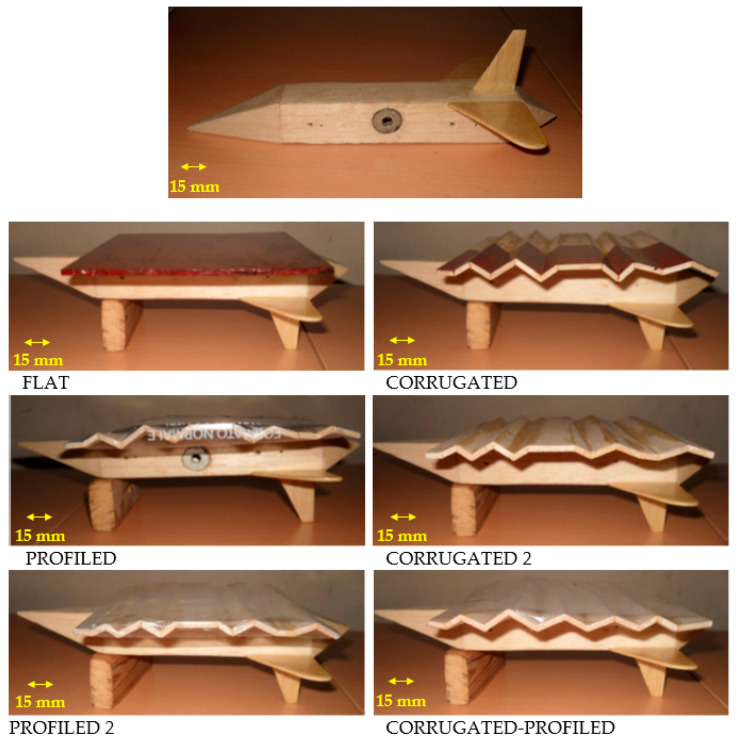
Details of MAV geometry and of six tested wing configurations.

In the present work, measurements have been made in a subsonic wind tunnel with a circular cross-section (diameter equal to 1 m) for a complete MAV model, having a square planform wing (chord and span equal to 150 mm, aspect ratio AR = 1) with different corrugation surfaces. Taking inspiration from investigations made by Kesel [4] and Murphy and Hu [11], the investigated geometries are a flat plate, two bio-inspired corrugated wings, the same wings covered with deformable polyethylene films, and a mixed corrugated–profiled wing, as detailed in Figure 1, where the dimensional details of the corrugations are also included. All wing configurations were mounted on a complete MAV prototype, including the nose, fuselage and tail surfaces (not corrugated), also shown in Figure 1. Wings were positioned at the bottom of the MAV, covering the entire fuselage except the front pyramidal nose. The first corrugated wing and the related profiled one had quite sharp corners, as those reported in [4,11]. On the other hand, the second corrugated and profiled wings had the same geometry as the first ones, but with smoothed corners and with wing thickness reduced by 21%, with the intention of delaying flow separation at larger angles of attack.

The mixed corrugated–profiled wing is profiled only on one side (the lower side, not in contact with the fuselage), with a corrugation geometry with similar geometry and performance as the first corrugated wing. This mixed configuration is inspired by numerical simulations at small Reynolds numbers (less than 10^4^) [13], where beneficial effects were observed using corrugations only on one side of the wing (note that models are flipped in Figure 1). Referring to profiled wings, polyethylene films are in tension but obviously not rigid, so they allow fluid flow to deform the wing surface.

The free stream velocity of the wind tunnel was changed between 8 m/s and 13 m/s to attain Reynolds numbers between 8 × 10^4^ and 1.3 × 10^5^. Two types of measurements have been performed, i.e., global lift and drag using force balance as well as velocity fields over the wing using particle image velocimetry (PIV).

A TecQuipment force balance was used for drag and lift measurements (D and L, respectively). The two forces are defined as those measured along streamwise direction, x, and vertical directions, y. The balance was connected to the model by means of a horizontal steel cylindrical bar, and in order to account for the contribution of this to the total measured drag, measurements without the model were performed (only the bar). This measurement was subtracted from the overall drag to determine the drag due only to the model, assuming an almost linear contribution. The force measurements for each test case were averaged five times, each one with a duration of around 10 s. Considering that a typical large-scale time of the phenomenon is around c/U_0_~10^−2^ s (c is the wing chord and U_0_ is the free stream velocity), the selected acquisition time interval ensures proper statistical convergence. The overall estimated uncertainty of the force balance measurement is ±2%. The drag and lift coefficients are defined as

CD=D12ρU02A, CL=L12ρU02A

where *ρ* is the air density at an ambient temperature and *A* is the wing surface area. It is important to consider that for a proper comparison between the results obtained by other authors on airfoils and the present results on full wings with a small aspect ratio, a correction due to finite wing aperture has to be applied [23]. Specifically, the effective angle of attack of airfoil data in the present plots, α*_eff_*, is obtained as the sum of the airfoil angle of attack, α_0_, plus the induced angle due to tip vortices, expressed in terms of the lift coefficient and aspect ratio, as

$$\alpha_{eff}=\alpha_{0}+\frac{C_{L}}{\pi AR}$$

Velocity field measurements over the wing surfaces were carried out via planar two-component particle image velocimetry (PIV) on a vertical plane at free-stream velocity around 10 m/s. The acquisition system is made up by a Quantel Nd-Yag double pulsed laser, with a 532 nm wavelength, 100 mJ per pulse, 7 ns pulse minimum duration, a cross-correlation PCO Pixelfly camera with resolution 1392 × 1040 pixels fitted with a 50 mm lens (aperture 1.2) and a BNC 575 synchronizing unit.

**Figure 2:**
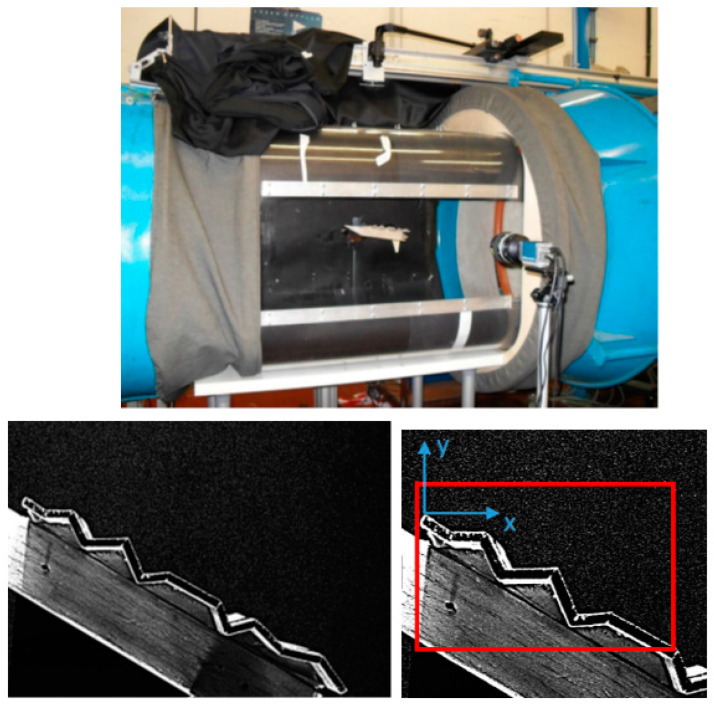
PIV set-up with laser arm, camera and MAV model in wind tunnel (**top**). Example of PIV-acquired images at full scale (large field of view) and reduced scale (small field of view, in red) with reference system (**bottom**).

A narrow band-pass filter (center wavelength—530 nm; bandwidth—10 nm) from Edmund Optics was added to the optics in order to filter reflections from wing surfaces, painted with Rhodamine coatings, which is also visible in the flat, corrugated and profiled models in Figure 1. The acquired vertical plane (x, y) spans the entire wing surface (around 150 mm, i.e., one chord) at the wing centerline. In a second test, a smaller portion of the wing close to the leading edge (around 80 mm) is considered with higher resolution. These are detailed in Figure 2, together with the overall measurement arrangement and reference system. The laser sheet thickness is approximately 1 mm.

The flow was seeded with oil droplets with an estimated mean diameter of 2 μm, resulting a Stokes time scale around 10^−4^ s, which is sufficiently small to resolve the large scale of flow field (as previously noted, the time derived from the wing chord divided by the mean velocity is around 10^−2^ s). Each acquisition included 2048 image pairs, and the acquisition frequency was 5 Hz, corresponding to an acquisition interval of more than 400 s so far. This is much larger than the previously reported typical large-scale time of the phenomenon, so it ensures statistical moment convergence. PIV software, DaVis 7.2 by LaVision Gmbh, Göttingen, Germany, has been employed for instantaneous fluid velocity field computation. The software relies upon an advanced image deformation multi-pass PIV cross-correlation algorithm with a window offset, adaptive window deformation and Gaussian sub-pixel approximation, thoroughly described in [24]. The minimum window size was set to 32 × 32 pixels with 75% overlapping, with a vector spacing of 8 pixels, corresponding to roughly 1 mm. The maximum displacement over two consecutive frames was equal to 13 pixels. The average estimated uncertainty of the measured velocity components is 3% with a 95% confidence level based on Student’s distribution.

## 3. Results

At first, the experimental results obtained for global forces acting on MAVs are presented, and then detailed velocity measurements are provided for those configurations that showed the best performance.

### 3.1. Global Force Measurements

**Figure 3:**
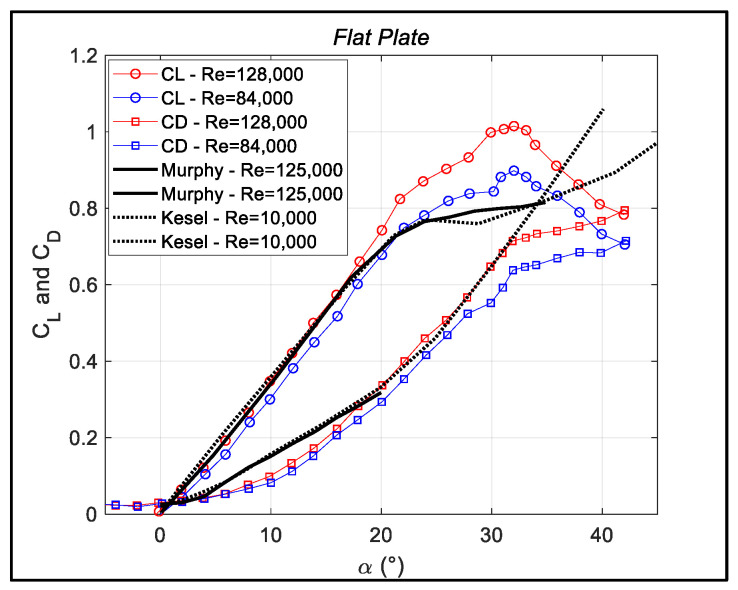
Lift and drag coefficients as functions of angle of attack for flat-plate MAV geometry. Present data are plotted for two Reynolds numbers and compared to data in [4,11].

The lift and drag coefficients obtained from the present measurements of an MAV equipped with a flat plate are shown in Figure 3, for two different Reynolds numbers. Regarding the lift coefficient, in the almost-linear region (α < 20°), there is only a slight dependence on the Reynolds number, as also confirmed by a comparison with reference results obtained at very different Reynolds numbers [4,11]. For a proper comparison with the present data, the reference data, obtained for an airfoil (AR → ∞) and for a finite wing (AR = 3.5), respectively, have been corrected to the effective angle of attack, as reported in the previous section. Also, note that all presented data refer to Reynolds numbers lower than those predicted for a full transition to a turbulent regime.

It is important to note that while there is good agreement between the present data and reference data in the linear part of the curve, differences appear when the angle of attack is approaching a stall (20° < α < 25°). For the present low-aspect-ratio MAVs, stall delay is a direct consequence of the presence of tip vortices in comparison to reference data. These data are attained for large-aspect-ratio wings, where no or strongly reduced tip vortices are present [25]. In addition, in the stall region, as expected, the results are much more Reynolds number-dependent. Concerning the drag coefficient, the present data approach a more parabolic behavior for a small angle of attack in comparison to the reference data. This is to be expected due to the fact that the latter refer to airfoils [11] or to slender wings [4].

**Figure 4:**
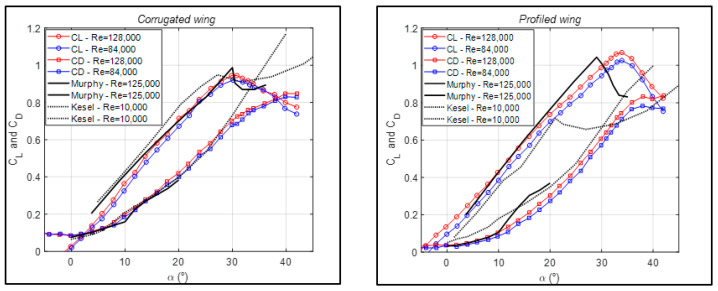
Lift and drag coefficients as functions of angle of attack for the corrugated and profiled MAV wing geometry. Present data are plotted for two Reynolds numbers and compared to data in [4,11].

For MAVs equipped with corrugated and profiled wings, results are provided in Figure 4. A comparison with the flat-plate configuration will be detailed in the following section, whereas here it is pointed out that for corrugated MAVs, Reynolds number dependence is even more attenuated, also at angles of attack close to stall. Even if the details of the corrugated geometries could be different (angles and lengths of linear elements), comparisons with data by Murphy and Hu [11] are rather good. For profiled wings, differences are larger, probably due to more relevant modifications of wing profiling between present and reference conditions (profiling material and degree of tension). To this end, it is important to stress again that the present wing profiling is simply obtained by covering corrugations with a deformable film. Again, stall delay is observed for the present low-aspect-ratio MAV wings in comparison to the high-aspect-ratio wings of the reference data.

**Figure 5:**
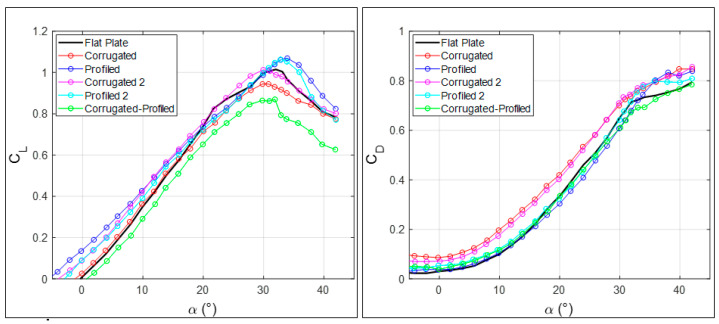
Lift and drag coefficients as functions of angle of attack for all tested MAV wing geometries for present large Reynolds number measurements.

Once comparisons with some of existing data have been established, a full comparison between all configurations that were tested in the present experiments is presented in Figure 5. Here, the lift and drag coefficients are given separately, to better highlight differences, only at the largest tested Reynolds number (Re = 1.3 × 10^5^), given the substantial Reynolds number independence observed in Figure 3.

For the lift coefficient (shown on the left in Figure 5), the best results in terms of the maximum lift coefficient and delayed angle of stall are obtained with the profiled configurations. The corrugated ones are closer to the flat-plate geometry, while the mixed corrugated–profiled configuration shows a drastic reduction. The improvements for profiled configurations are around 0.05 in lift coefficient at α = 10° and around (2–3°) in stall angle at the maximum lift coefficient. As already reported in Figure 4, these results are in good agreement with results presented in [11] at similar Reynolds numbers, but not so much with those in [4] at much smaller Reynolds numbers.

Regarding drag (on the right in Figure 5), the best performance is related to minimum drag coefficients. Corrugated configurations without any profiling are again ruled out, whereas all profiled configurations, including the mixed one, show drag coefficients similar to or lower than the reference flat-plate configuration. Therefore, it seems that the beneficial effect in comparison to a standard wing configuration is mostly obtained due to wing surface modifications due to elastic profiling, rather than to surface large-scale roughness, due to corrugations.

**Figure 6:**
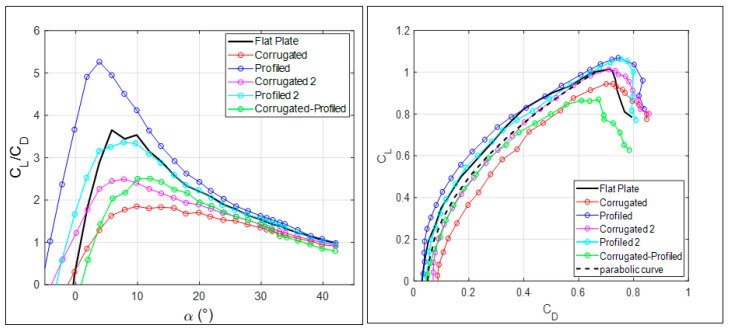
Lift-to-drag ratio and polar curve for all tested MAV wing geometries.

To summarize the performance of MAVs equipped with different wings, the ratio between the lift and drag coefficients and the so-called polar curves are reported in Figure 6. In agreement with previous findings, the lift-to-drag ratio, which is also related to wing efficiency in aerodynamics, is definitely superior, more than 1.5 times, for the MAV model equipped with profiled wings. In comparison to the flat-plate configuration, the smoothed profiled corrugation (Profiled 2) also attains good performance. All corrugated wing configurations, including the mixed one, have very poor performance, even in comparison to the flat plate. The polar curves, also reported in Figure 6, show that the data are close to a parabolic curve in all conditions, which is also indicated in the figure. This is a direct indication of the important effect of tip vortices for the present low-aspect-ratio wings. Indeed, tip vortices induce a dependence of the drag coefficient on the square of the lift coefficient, as obtained from finite wing theory [25]. Minor deviations from parabolic behavior are due to deviations from the isolated wing configurations, as in the present measurements where whole MAV vehicles are considered.

By considering the amount of measured differences between the geometries shown in Figure 5 and Figure 6, it is important to point out that the present results establish the improved performance of profiling over corrugation, regardless of the specific details used in corrugated and profiled wings. So far, results regarding global force indicate that the major advantage obtained from the tested MAV wing configurations is related to the effect of deformation of the wing under local fluid phenomena, so that the local wing shape proves to be more important than local roughness. Detailed velocity measurements over the wing were performed to clarify this aspect.

### 3.2. Local Velocity Measurements

As previously reported, detailed velocity measurements are provided only for the following: the reference flat-plate configuration, the profiled configuration with the best performance and the corrugated configuration. Velocity tests have been performed at a Reynolds number equal to Re = 10^5^.

**Figure 7:**
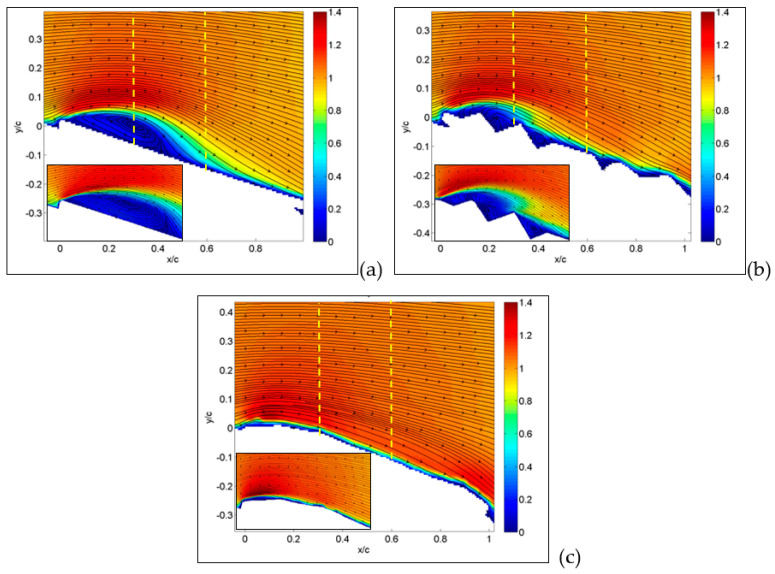
The average absolute value of velocity and streamlines at an angle of attack of 15° for MAVs with the following wing configurations: flat (**a**), corrugated (**b**) and profiled (**c**). Large field of view. In the insets, detailed small-field-of-view plots are reported. Dashed lines indicate positions for velocity profiles.

The first result deals with an angle of attack equal to 15° for the large-field-of-view image, as reported in Figure 7, where absolute values of velocity and streamlines are presented. This angle of attack is in the middle of the region where the lift coefficient is almost linear and the drag coefficient is nearly parabolic. In these conditions, the MAV with a flat plate (a) already shows a separation region starting from the wing leading edge, indicated in dark blue in the figure. As expected, over the upper part of the wing surface, there is an increase in velocity in comparison to free-stream conditions.

This view is confirmed in the inset, obtained from measurements on a smaller field of view, i.e., with a higher resolution. The origin of separation is clearly related to the flat plate leading edge.

For the corrugated case (b), the separation is significantly reduced in comparison to the flat plate, and velocity decrements are observed in cavity regions over the upper part of the wing. The inset shows how recirculation is generated in each cavity of the corrugated wing. For the MAV with a profiled wing, even in the detailed flow view, no separation and no relevant reductions in velocity over the wing surface were observed, except for the boundary layer close to the surface.

**Figure 8:**
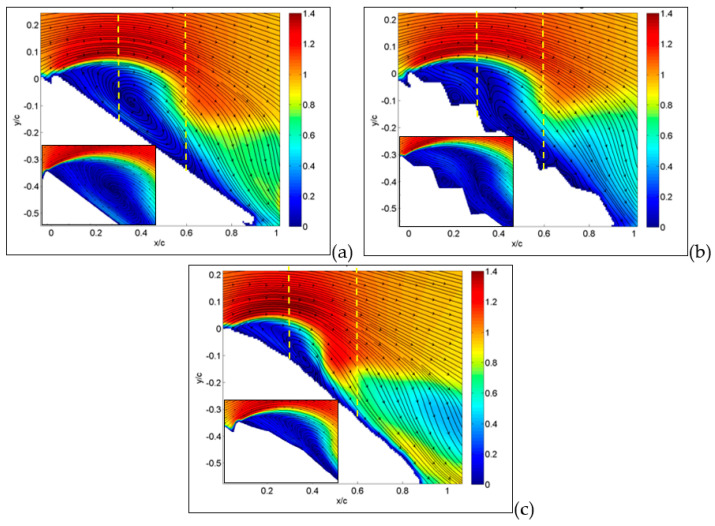
Average absolute value of velocity and streamlines at angle of attack of 30° for flat (**a**), corrugated (**b**) and profiled (**c**) MAV wing configurations. Large field of view. In the insets, detailed small-field-of-view plots are reported. Dashed lines indicate positions for velocity profiles.

The situation is quite different when the angle of attack is increased up to 30°, which is the angle of stall for corrugated configuration. As shown in Figure 8a,b, for the MAV with flat and corrugated wings, large separations are now clearly visible, extending over almost the entire upper surface, in a similar way for the two.

However, as recovered by the inspection of the small portions of the leading edge, while there is only a major large separation in the case of the flat plate, there are several recirculation regions in the case of the corrugated wing, related to each cavity. It seems that these are responsible for the large part of separation.

**Figure 9:**
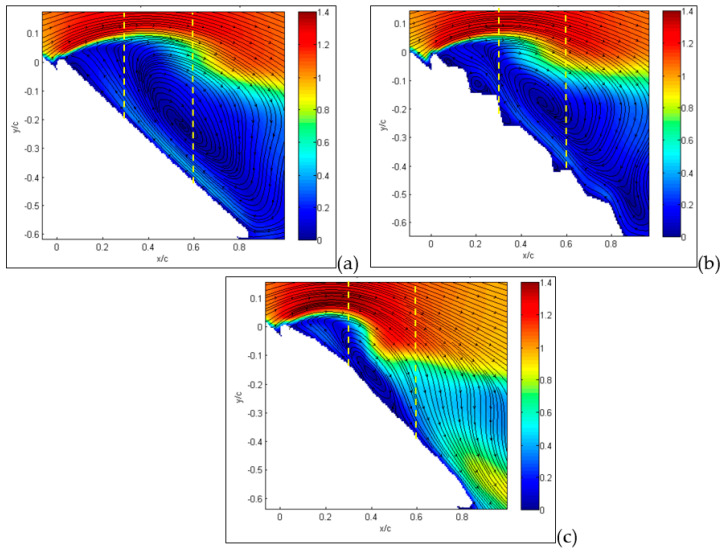
Average absolute value of velocity and streamlines at angle of attack of 36° for flat (**a**), corrugated (**b**) and profiled (**c**) MAV wing configurations. Large field of view. Dashed lines indicate positions for velocity profiles.

On the other hand, the separation is only at the starting level for the MAV with a profiled wing, as observed in Figure 8c. Moreover, there is a clear reattachment of the fluid around one half of the chord, which indicates a partial recover of lift, as reported in Figure 5. This situation is also almost unchanged up to an angle of attack of 36°, shown in Figure 9, where the flow field is fully separated for the flat-plate and corrugated configurations, while being quite largely separated for the profiled case. In this last case, there is still a small portion of the wing’s upper surface (almost 25%) with flow still attached, still giving some lift so far.

**Figure 10:**
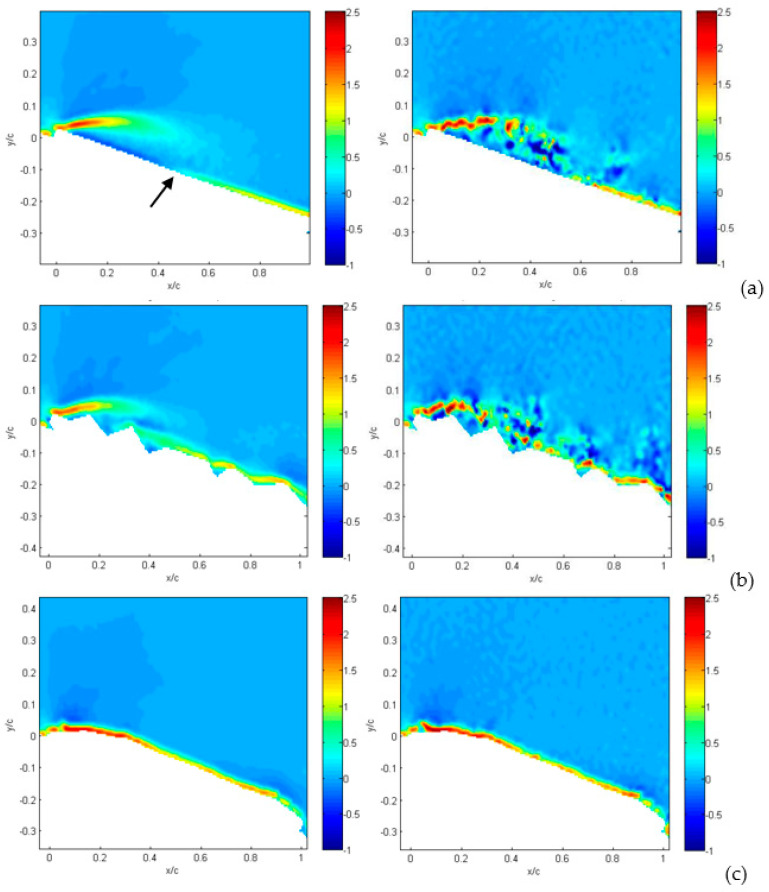
Average (on the left) and instantaneous (on the right) vorticity for an angle of attack equal to 15°: flat-plate (row **a**), corrugated (row **b**) and profiled (row **c**) MAV configurations from top to bottom. Large field of view.

In order to obtain information on the nature of the separation regions, detailed instantaneous and average vorticity maps are presented in Figure 10, for an incidence angle equal to 15° and for a large field of view. For each MAV configuration, the average vorticity map (on the left) and one instantaneous plot (on the right) are shown, so that it is possible to derive how the average is obtained from moving instantaneous vortices.

Considering the flat plate (Figure 10a), on average, it is possible to observe a well-defined layer, detaching exactly from the plate leading edge. By looking at the instantaneous plot, it is noticed how this layer is composed by clockwise vortices (in red) generated at the leading edge, over several counter-clockwise vortices (in blue) close to the flat plate surface. This sequence generates an adverse layer in the average plot (in blue), which is maintained down to the reattachment point, where the attached boundary layer growth restarts (in red, indicated by the black arrow).

For the corrugated wing, at this angle of incidence, the situation is similar, but as noticed, the presence of corrugation enables the reduction in the vorticity layer and the generation of less organized instantaneous clockwise and counter-clockwise vortices. This reduces the extension of the adverse layer and of the separation region, being replaced by a sequence of vortices in the wake of each corrugation.

**Figure 11:**
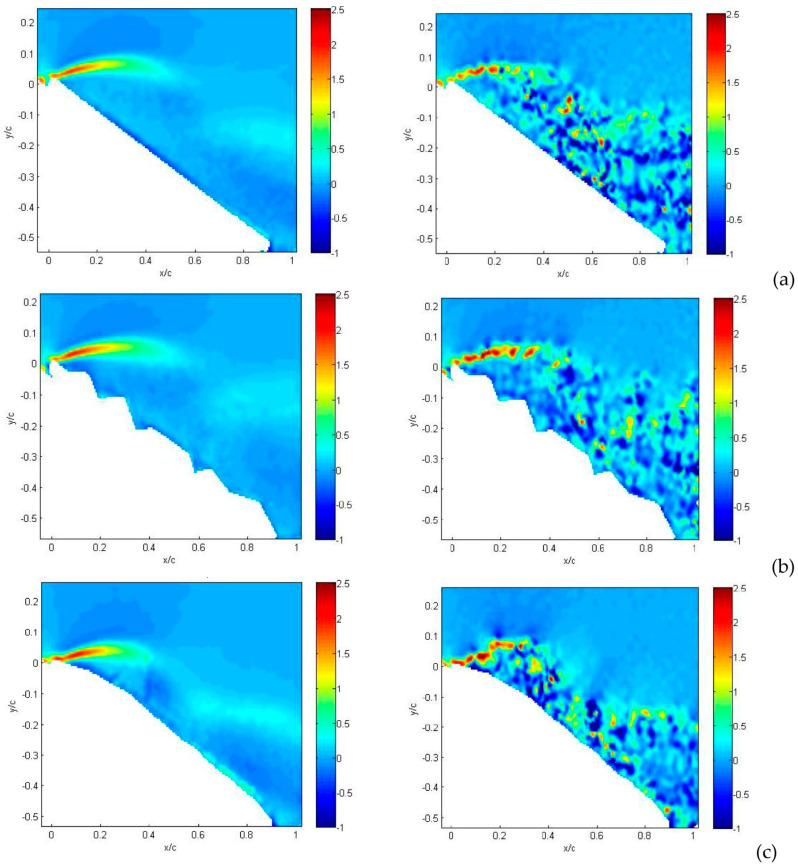
Average (on the left) and instantaneous (on the right) vorticity for an angle of attack equal to 30°: flat-plate (row **a**), corrugated (row **b**) and profiled (row **c**) MAV configurations from top to bottom. Large field of view.

When the wing is profiled, as in the third MAV configuration, the vorticity layer is fully attached to the curved upper surface, without separation and with no individual vortices in the instantaneous plot. The same analysis is repeated for an angle of incidence equal to 30°, as shown in Figure 11. The first two cases, the flat plate and corrugated wing (Figure 11a,b), point out a larger extension of the upper vorticity layer in the average field in comparison to the situation at a smaller angle of attack. In addition, a larger number of counter-clockwise vortices is observed in the instantaneous plots. So far, the overall picture confirms that separation takes place over the entire wing upper surface. From this point of view, the flow dynamics over the flat plate and the corrugated wing are not different. On the other hand, the profiled wing still shows a limited vorticity layer extension in comparison to the other two configurations. Vortex reattachment over the wing upper surface is attained around 50% of the chord, similarly to the other two configurations at a lower angle of incidence.

**Figure 12:**
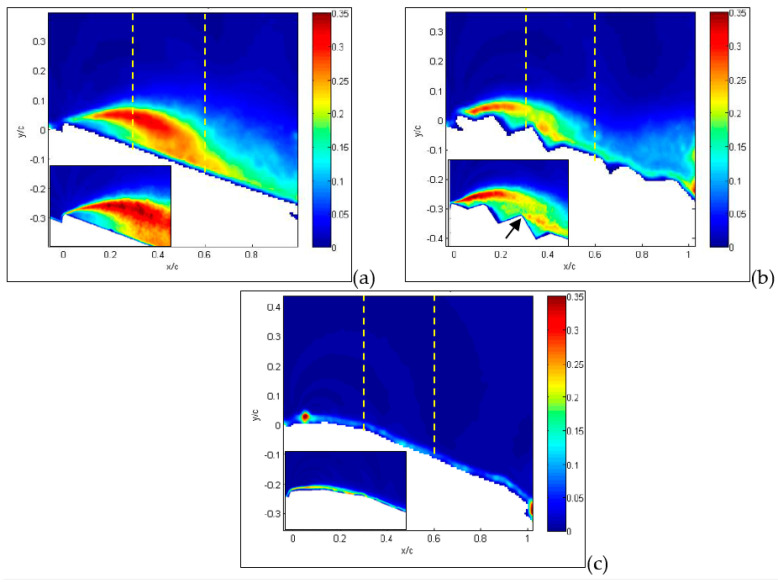
Average *rms* of streamwise velocity component for angle of attack equal to 15°: flat-plate (**a**), corrugated (**b**) and profiled (**c**) MAV configurations. Large field of view. In insets, detailed small-field-of-view plots are reported. Dashed lines indicate positions for *rms* profiles.

**Figure 13:**
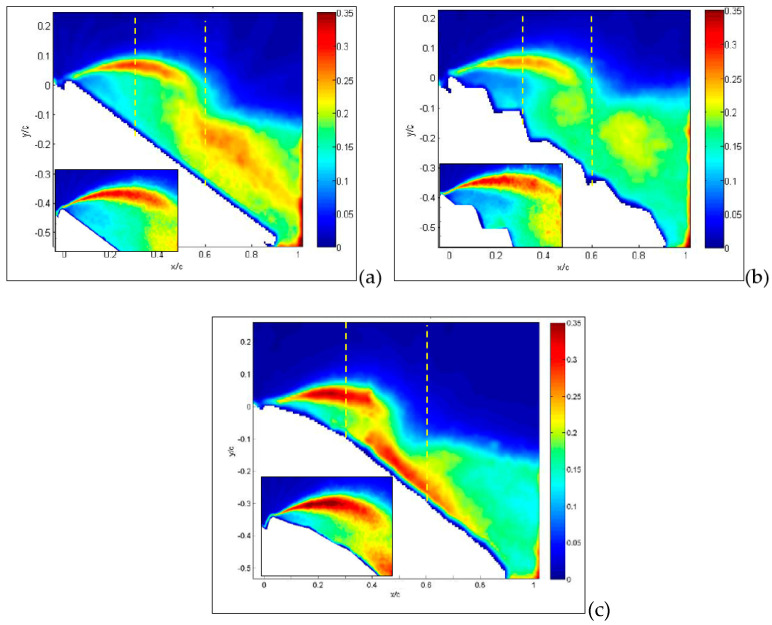
Average *rms* of streamwise velocity component for angle of attack equal to 30°: flat-plate (**a**), corrugated (**b**) and profiled (**c**) MAV configurations. Large field of view. In insets, detailed small-field-of-view plots are reported. Dashed lines indicate positions for *rms* profiles.

The last quantity to be considered from velocity measurements is the *rms* of velocity retaining information on turbulent fluctuations. The contribution of each velocity component to such fluctuations is similar, so they are presented together in Figure 12. For the flat plate, shown in Figure 12a, the wing leading edge acts as a sharp turbulence generator, i.e., an abrupt change in geometry that stimulates large turbulence fluctuations all over the separation region. For the corrugated condition, shown in Figure 12b, turbulence generation also includes the first corrugation and is overlapped with sharp turbulence generation over the second corrugation, as indicated by the arrow in the small-field-of-view enlargement. The interaction between these two large fluctuation regions allows flow reattachment on a shorter distance in comparison to the flat plate. On the other hand, no major generation of turbulence is obtained for the profiled wing, as shown in Figure 12c. When the angle of attack is increased up to 30°, as shown in Figure 13, this phenomenon is even more evident, but in this case, turbulence generation from corrugations is not sufficiently strong to interact with that from the leading edge. Therefore, there is no reattachment for corrugated and flat-plate configurations, as shown in Figure 13a,b, whereas it is observed for the profiled condition, as shown in Figure 13c.

The reason the profiled configuration still performs so well at such a large angle of incidence is that the deformation and oscillations of the profiled surface generate increased turbulence intensity (larger than 30%) in comparison to other two cases. This reduces the energy of the incoming flow, being converted to turbulence, so it allows flow reattachment—even at this large incidence angle—and the deviation of turbulence production towards the wing surface.

**Figure 14:**
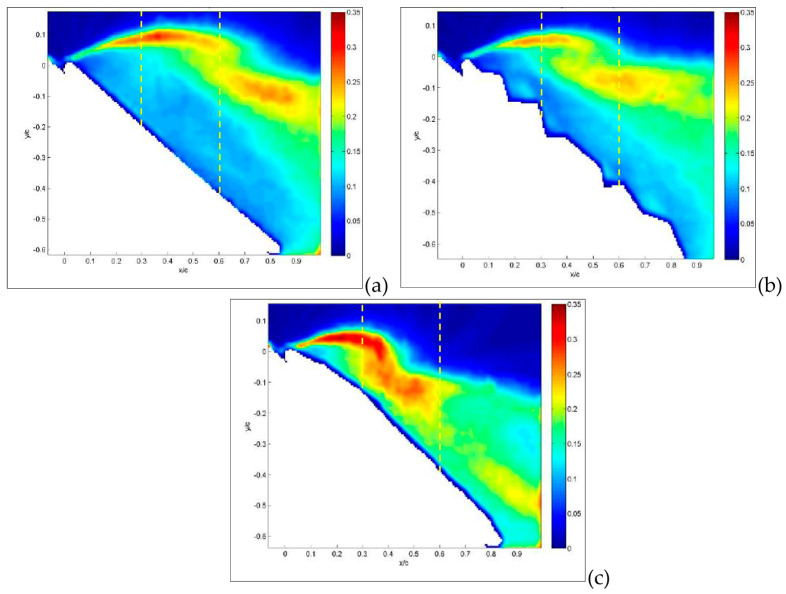
Average *rms* of streamwise velocity component for angle of attack equal to 36°: flat-plate (**a**), corrugated (**b**) and profiled (**c**) MAV configurations. Large field of view. Dashed lines indicate positions for *rms* profiles.

It is interesting to see that for an angle of incidence as large as 36°, as presented in Figure 14a,b for the flat plate and corrugated wings, turbulence production is moved very far from the wing upper surface. This indicates that complete flow separation occurs for these conditions. On the other hand, for the profiled wing, as shown in Figure 14c, the region of large turbulence fluctuations is still close to the wing upper surface, allowing a small amount of flow reattachment on the wing surface.

**Figure 15:**
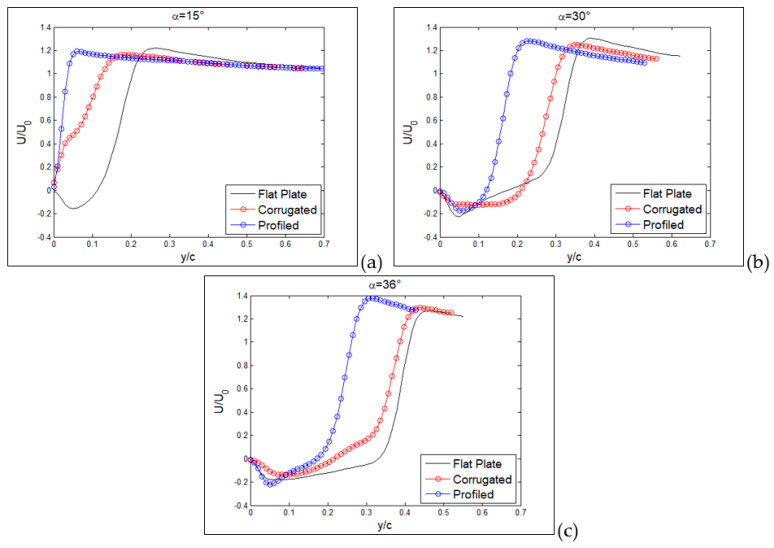
Vertical profiles of normalized streamwise velocity component at x/c = 0.3, for the three tested MAV configurations. Angles of incidence equal to 15° (**a**), 30° (**b**) and 36° (**c**).

From previous plots, global information has been derived, whereas more detailed comparisons can be made by considering profiles along a line, as reported in the following section. Hereafter, profiles are taken along the vertical direction as in the yellow dashed lines plotted in Figure 7, Figure 8 and Figure 9. So far, in Figure 15, profiles of normalized streamwise velocity at a distance from the leading edge equal to x/c = 0.3 are compared for the three tested MAV configurations and for angles of incidence equal to 15°, 30° and 36°. In these plots, consider that position y/c = 0 corresponds to the wing surface, where velocity must vanish, whereas moving to the right corresponds to increasing distance from the surface.

At 15° (Figure 15a), only the MAV equipped with a flat plate already shows a separation region with negative streamwise velocities, with the other two showing more or less steep profiles with positive velocities (no separation). At an angle of 30° (Figure 15b), all three profiles show separation, though it is less extended for the profiled configurations in comparison to the other two, which are quite similar. The last plot at 36° confirms this picture, also indicating similarity between the three, so that it is possible to state that separation for all conditions originated in the form of a leading-edge stall (stall from the initial part of the wing).

**Figure 16:**
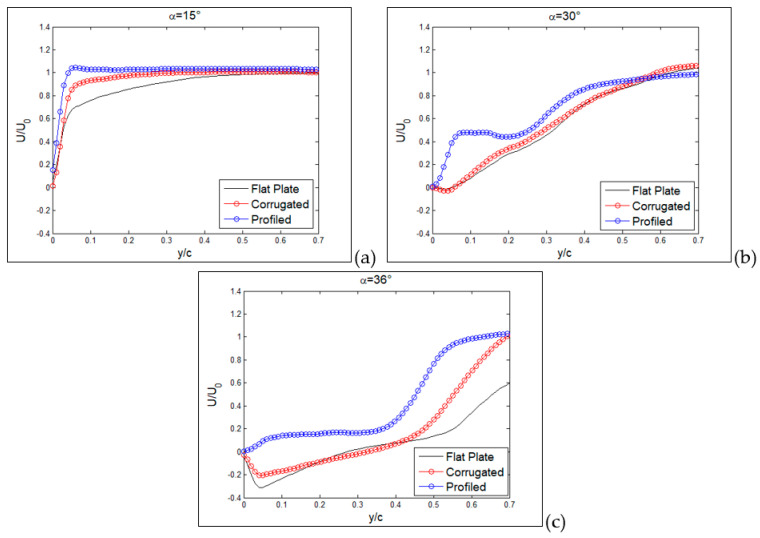
Vertical profiles of normalized streamwise velocity component at x/c = 0.6, for the three tested MAV configurations. Angles of incidence equal to 15° (**a**), 30° (**b**) and 36° (**c**).

In Figure 16, this comparison is reported at a distance from the leading edge equal to x/c = 0.6. Also, profiles at this position are indicated by yellow dashed lines in Figure 7, Figure 8 and Figure 9. From previous results, at this position, separation is expected only around stall angles or larger. Indeed, at an angle of incidence equal to 15° (Figure 16a), all three configurations presented a large positive velocity gradient, thus confirming that separation and related recirculation still did not reach this position and that the flow was still attached here. For an angle equal to 30° (Figure 16b), a small recirculation appeared for the corrugated and flat-plate configurations, whereas the profiled one still exhibited a positive gradient followed by extended regions of almost constant velocity, equal to around 0.4–0.5 times the free stream velocity. This last behavior indicates that for the profiled configuration, the external flow is not trapped in a recirculation region but still flows over the wing surface, even if not as efficiently as in pre-stall conditions. For an angle of incidence equal to 36° (Figure 16c), corrugated and flat-plate data show separation over large distances from wing surface. Again, they followed a similar behavior, whereas the profiled configuration remained attached, with a constant-velocity region extending at a large distance from the wing surface.

**Figure 17:**
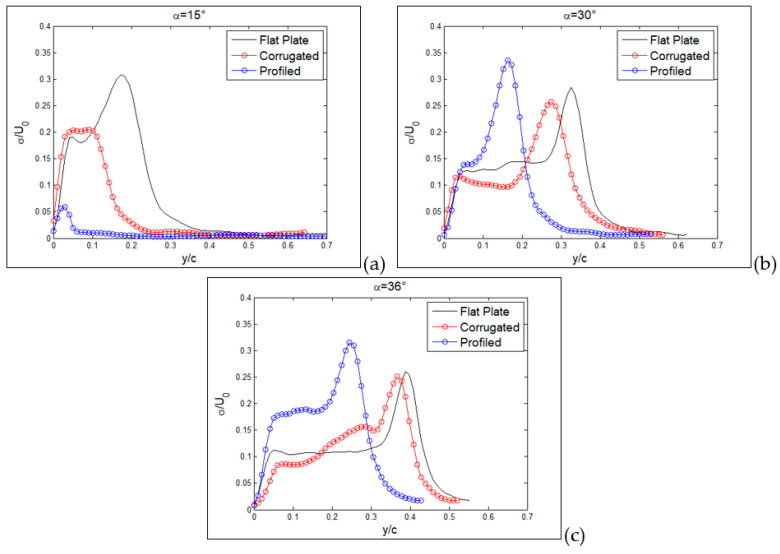
Vertical profiles of normalized *rms* streamwise component at x/c = 0.3, for the three tested MAV configurations. Angles of incidence equal to 15° (**a**), 30° (**b**) and 36° (**c**).

**Figure 18:**
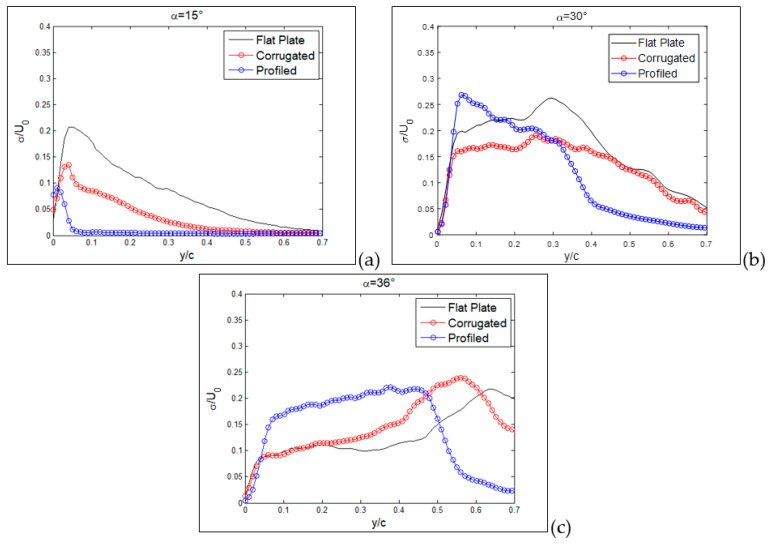
Vertical profiles of normalized *rms* streamwise component at x/c = 0.6 for the three tested MAV configurations. Angles of incidence equal to 15° (**a**), 30° (**b**) and 36° (**c**).

The detailed vertical profiles of *rms* streamwise velocity are reported in Figure 17 and Figure 18 at the same axial distances and incidence angles as in the last two figures. Yellow dashed lines indicate the positions of these vertical profiles in Figure 12, Figure 13 and Figure 14. For the flat plate, at α = 15° (Figure 17a), fluctuations are very large (over 30%) in a region close to the wing surface (y/c = 0.2 for the flat plate). This is just the region where a layer formed by moving small-scale vortices is present. A similar situation is observed for the corrugated MAV, albeit with smaller intensity. For the profiled condition, only a small increase in *rms* is observed towards the wing surface (around 5%). This indicates that no production of turbulence is present in this case. When the angle of incidence is increased, as shown in Figure 17b,c, the situation is very similar between the three configurations. High fluctuations (over 25%) are observed at different distances from the wing surface, increasing with the angle of incidence and depending on the specific configuration. The flat-plate and corrugated configurations are more and more similar as the angle of attack increases.

When profiles are compared at a distance x/c = 0.6 and a small angle of incidence, as shown in Figure 18a, all *rms* profiles are equivalent to those observed close to solid walls, i.e., a continuous increase in turbulence intensity for decreasing wall distance and then a rapid decrease to zero at the wall (y/c = 0), indicating almost no separation. At an angle of 30°, as shown in Figure 18b, the peak due to separation in the external region starts to appear in the flat-plate and corrugated configurations, whereas the profiled one still keeps an almost regular increase towards the wall, as in the case of no separation. Lastly, at 36°, as shown in Figure 18c, the separation extends all over the investigated region for all configurations.

## 4. Discussion and Conclusions

This work focuses on the aerodynamics of micro air vehicles (MAVs) equipped with bio-inspired wings. Starting from observations of wings from dragonflies, butterflies and locusts, which present rigid elements connected by flexible parts, corrugated wings were considered in several configurations, also using flexible plastic covering for profiling. While previous studies on the argument were mostly focused on isolated wings, with high aspect ratios, here entire MAVs with low-aspect-ratio wings were investigated. Comparisons were made with a classical flat-plate configuration, with a rigid surface and with previous investigations on corrugated wings. The use of a planform with a low aperture-to-chord ratio allows investigating how the effect of a finite wing, with the related presence of large tip vortices over the whole wing, was coupled with surface bio-inspired geometries.

The global performance of each model was obtained by measuring the drag and lift coefficients using a dynamometric balance, and they were coupled to local detailed velocity fields over the wing surfaces using particle image velocimetry (PIV). All measured quantities were averaged in time, and instantaneous velocity fields were also considered.

The main comments derived from the present measurements are the following:

- In the classical flat-plate configuration, the present force coefficients compared well with those of other authors, even if they presented some difference in the Reynolds number. The main advantage of the present configuration is the beneficial effect of large tip vortices over the entire wing, which allows for delaying stall over more than 30°, with only slight increases in drag. This is due to the small aspect ratio used for the present wings.
- The present MAV, equipped with corrugated surfaces, attained similar performance and stall angles as previous investigations on similar geometries, even if those used different planforms. On the other hand, the profiled configuration, i.e., the one with corrugations covered by a flexible surface element, was able to delay stall to angles larger than 30°, i.e., more than in previous investigations. No relevant increases in drag were measured for the present configurations. Again, the beneficial effects of low-aspect-ratio wings were responsible for this.
- Among all tested configurations, having different corrugation and profiling geometries, the best is a simple flexible covering joining all corrugations, avoiding abrupt changes in the local surface shape. This could raise the efficiency of the wing, i.e., the ratio between lift and drag, even by a factor 1.5. On the other hand, simple corrugation is not suitable to achieve significant improvements in comparison to a flat plate, due to the occurrence of large-scale separation at the leading edge. This is in agreement with some recent studies [17].
- The phenomena contributing to global MAV performance are related to the formation of large flow separation, partially attenuated by the combined effect of cavities and elastic profiling. In the present tests, these are present in almost all conditions when the angle of attack is increased. However, for the profiled configuration, after separation, there is a flow reattachment over the wing surface, which allows reaching a maximum angle of incidence as large as 36°. This is mostly due to the beneficial effect of large-scale tip vortices, present in these small-aspect-ratio wings, which were not present in the high-aspect-ratio wings used in previous investigations [5,11].
- The separation region is bordered by lower-scale vortices detaching from the wing surface at the leading edge, thus generating shear layers, characterized by high vorticity levels and high turbulent intensity fluctuations, even as large as 30%.The details of the separation region extension and velocity profiling are revealed very well by detailed high-resolution velocity fields. They allow individuating specific points where reattachment takes place for the profiled configuration.

From the present measurements, it is clear that only the combination of wing corrugation and profiling with a flexible surface can give effective advantages in present MAV performance. In other words, the use of corrugated wings alone cannot give substantial increments in performance in comparison to the flat-plate configuration.

The main limitations of the present work are the reduced number of corrugation geometries tested here, which surely can be improved by also looking at corrugations inclined in respect to the mean flow and by looking at fully 3D corrugations. However, previous investigations already selected the best configurations in terms of geometrical construction and operative performance [5,8,11,14,17], so this point is not primarily relevant. On the other hand, a relevant extension of this work is the investigation of planforms different from the rectangular one, as well as the specific study of different wing sections.

In parallel, high-speed investigations could detail unsteady flight, instantaneous behaviors in time and possible surface deformations derived from instantaneous velocity fields, and they could compute instantaneous loads, also considering the effect of wing flapping and/or sliding. This last point is surely the next step of this research project.

## Data Availability

Data are available on request to the authors.
